# Economic Analysis of the Use of VCS2000 for Pork Carcass Meat Yield Grading in Korea

**DOI:** 10.3390/ani11051297

**Published:** 2021-04-30

**Authors:** Juntae Kim, Hyo-Dong Han, Wang Yeol Lee, Collins Wakholi, Jayoung Lee, Youn-Bok Jeong, Jeong Hwan Bae, Byoung-Kwan Cho

**Affiliations:** 1 Department of Biosystems Machinery Engineering, College of Agricultural and Life Science, Chungnam National University, 99 Daehak-ro, Yuseoung-gu, Daejeon 34134, Korea; biosch@o.cnu.ac.kr (J.K.); wcoln@o.cnu.ac.kr (C.W.); jaylee91@o.cnu.ac.kr (J.L.); 2 Korea Institute for Animal Products Quality Evaluation, 21 Areumseo-gil, Sejong 30100, Korea; hhdong@ekape.or.kr (H.-D.H.); iling@ekape.or.kr (W.Y.L.); apgs0122@ekape.or.kr (Y.-B.J.); 3 Department of Applied Animal Science, Kangwon National University, Chuncheon 24341, Korea; 4 Department of Economics, Chonnam National University, Gwangju 61186, Korea; jhbae@chonnam.ac.kr; 5 Department of Smart Agriculture Systems, College of Agricultural and Life Science, Chungnam National University, 99 Daehak-ro, Yuseong-gu, Daejeon 34134, Korea

**Keywords:** VCS2000, pork carcass meat yield grading machine, economic analysis, NPV, sensitivity analysis

## Abstract

**Simple Summary:**

Koreans consume more pork meat among the various meats, and as the consumption of pork meat increases, the amount of slaughtering is also increasing. Accordingly, the Korean government is sizing up and modernizing slaughterhouses to expedite the slaughter and to increase the safety of livestock products. The labor-intensive slaughter industry is undergoing major changes due to the COVID-19 pandemic, which will speed up the automation of slaughterhouses. Various automation devices are also being introduced in Korea, and currently the Korean government is introducing a pork meat yield grading machine to increase the accuracy of judgments based on the increasing slaughter volume. However, because such equipment is quite expensive, it is not easy to introduce, and a detailed study on the utility of the related equipment and an economic feasibility analysis has not been conducted. Therefore, in this study, we tried to prove the validity of the introduction of the equipment through the effectiveness study and economic analysis of the automatic meat yield grading machine, and a plan to increase the economic effect was considered.

**Abstract:**

Currently, the pork industry is incorporating in-line automation with the aim of increasing the slaughtered pork carcass throughput while monitoring quality and safety. In Korea, 21 parameters (such as back-fat thickness and carcass weight) are used for quality grading of pork carcasses. Recently, the VCS2000 system—an automatic meat yield grading machine system—was introduced to enhance grading efficiency and therefore increase pork carcass production. The VCS2000 system is able to predict pork carcass yield based on image analysis. This study also conducted an economic analysis of the system using a cost—benefit analysis. The subsection items of the cost-benefit analysis considered were net present value (NPV), internal rate of return (IRR), and benefit/cost ratio (BC ratio), and each method was verified through sensitivity analysis. For our analysis, the benefits were grouped into three categories: the benefits of reducing labor costs, the benefits of improving meat yield production, and the benefits of reducing pig feed consumption through optimization. The cost-benefit analysis of the system resulted in an NPV of approximately 615.6 million Korean won, an IRR of 13.52%, and a B/C ratio of 1.65.

## 1. Introduction

As the consumption of pork increases in Korea, the number of pigs raised and slaughtered is also increasing. As of 2018, the per capita meat consumption in Korea was 53.9 kg, and pork consumption was 27 kg, about 50% of the total meat consumption, making it the most consumed meat [1]. The total number of pigs raised in Korea also increased, reaching a new high of 11.64 million in 2018, up 1.3% from the previous year [1]. As of 2018, the number of pig farms with fewer than 1000 head fell 2.4% year-on-year, while the number of breeding pigs per farm was 1879, up 3.8% from the previous year. Similarly, the number of slaughtered pigs increased from 15 million in 2014 to 17 million in 2018 [2].

To improve the quality and consistent supply of livestock products, domestic slaughter facilities are being modernized to scale up production. Many slaughterhouses globally currently employ the “on-the-rail system”, which aids in the continuous movement (linear and rotation) of the carcasses using electronic controls. This system has the ability to handle between 600 and 1000 carcasses per hour [3]. However, most mainstream slaughterhouses in Korea are capable of handling approximately 300 pigs per hour. To accelerate slaughterhouse throughput, the Korean government has designated pathways to modernize slaughterhouses (i.e., by improving facilities and standardizing sanitation procedures) and establish them as base slaughterhouses [4]. According to data from the Korea Livestock Quality Evaluation Institute, as of 2016 four slaughterhouses existed that slaughtered about 400 head of pigs per hour or more, and three slaughterhouses that slaughtered more than 300 head. In the case of a scaled slaughterhouse, a pig carcass must be graded within 12 s based on 300 head/h and 9 s based on 400 head/h to handle the slaughter without delay [4].

Korean domestic pork carcass grading is determined based on 21 parameters, including gender, carcass weight, back-fat thickness, appearance, and meat quality and defects. Currently, the conventional grading method consumes a substantial amount of time because it is based on manual measurements and judgements. The measurement and grading results are subjective and vary depending on the grade judge, for whom training also requires a significant amount of time and money. The scale of slaughtering facilities leads to an increase in slaughter volume, which can increase the fatigue of judges and decrease the accuracy of measurement due to an increase in the number of carcasses. Continued accumulation of judging errors can lead to economic losses for livestock farms and affect the reliability of livestock product quality. Therefore, to increase the accuracy of the scaled slaughterhouse’s grading results and minimize the manpower input, the Korea Livestock Quality Evaluation Institute has promoted a pilot project to introduce and utilize automatic grading machines since 2016. The VCS2000 (e+v Technology GmbH & Co.KG, Oranienburg, Germany), an automatic grading system for pig carcasses, was introduced as a government project.

**Figure 1:**
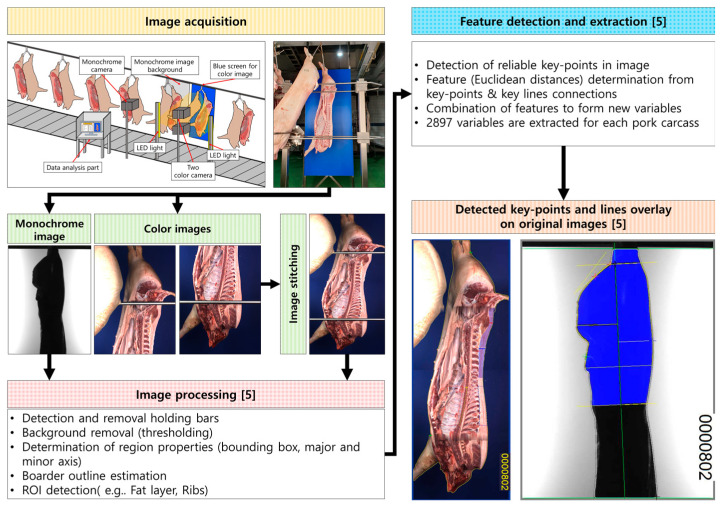
Summary of image processing and feature selection for the VCS2000 system [5].

The VCS2000 system consists of three main parts (automatic grading machine, radio-frequency identification (RFID) system, and carcass number printer). The VCS2000 uses two color cameras and one binary camera for grading and meat yield analysis. The system is capable of predicting about 52 parameters of pork carcasses through image analysis (Figure 1). Finally, the machine determines the total lean meat percentage (LMP), prime cuts LMP (seven parts), and ‘caky-fatty’ pork belly information. With the use of automatic grading machines, the 21 items currently judged by existing personnel can be expanded to 52 items. Moreover, grading can be expected to provide more precise judgments and can thus be used as an accurate tool for improving pig breeding and reducing feed costs.

Although the VCS2000 core analysis algorithm is not disclosed because it is proprietary company technology, results from the use of the system are relatively easy to establish. In predicting the LMP of Spanish pork carcasses using the VCS2000, R^2^ and RMSE results were reported to be 0.70 and 2.3%, respectively [6]. In addition, the results of the LMP data from the VCS2000 have been used to correlate the genetics and meat of the pig breed [7,8]. Studies related to VCS2000 pork meat yield grading machines include the development of yield prediction models and the status of system operation for Korean pork carcasses. In a study of the performance of the system for prediction of Korean pork carcass yields, the resultant formula was able to achieve an R^2^ of 0.77 and the root mean square error (RMSE) for predicting the LMP for the whole carcass was 2.12%. Furthermore, R^2^ values for ham, belly, and shoulder cuts were reported to be above 0.8; these results are superior to results from the European pork carcass LMP formula developed using the same VCS2000 system [5].

Cost-benefit analysis is a typical method used for economic assessment [9]. This tool allows for the economic value of a project to be assessed before the start of the project or at the stage of the project to be justified. Cost-benefit analysis compares all possible social costs and social benefits to their currency value, and when the “benefits” arising from the project are greater than the “costs” incurred to achieve the project goal, the project is deemed to be valid from a business perspective [9]. Although cost-benefit analysis has the disadvantage of being limited to a small number items that can quantify benefits and costs, it is an appropriate method for evaluating public investment projects because there is little room for the evaluator’s involvement in the analysis process and it can be compared on a uniform scale. Regarding the livestock industry, cost-benefit analysis has been used to analyze the costs and benefits of improving pig breeding facilities to reduce their odor [10]. At the beginning of the introduction of hazard analysis and critical control points (HACCP), cost-benefit analysis was used to clarify the effects and costs for the meat and milk processing industries [11,12]. Case studies have been recently under taken in which this analysis was used to assess the economics of a complex division automation system that can automate splitting and spinal cord removal [13].

Because slaughterhouses are currently undergoing modernization globally there is a high demand for automation. Thus, automated devices, such as automatic meat yield estimation and grading equipment, will continue to be introduced. However, few studies have been conducted on the economic impact of the introduction of such expensive automation devices. Therefore, this study was conducted to determine whether this equipment could have a positive effect on the Korean livestock industry by analyzing the economic effects of the introduction and use of VCS2000, an automatic grading system for pig carcasses.

## 2. Materials and Methods

Cost-benefit analysis was conducted through a series of processes for determining project periods, detecting benefits and costs of the given project, determining the discount rate, economic assessment, and sensitivity analysis (Table 1).

### 2.1. Decision of Project Periods

When determining the life of a project, it is necessary to determine the extent to which the business is expected to continue. The sustainability of the project is determined by the functions of the target and is decided based on the life of the target. Project life is divided into physical and economic life; physical life refers to the period after which a facility is aged and unable to perform its original function, and the economic life (persistent period) is the period after which the facility’s economic value has been reduced to zero. The Public Procurement Service (PPS) set the persistent period of the pork meat yield grading machine (PPS category number: 2110191501) to 9 years [14], and thus the project life of the machine was set to 9 years in this study.

### 2.2. Setting of Discount Rate

Discount rate refers to the rate used to convert future values into a present value, which is the most important criterion for evaluating the return of the project in cost-benefit analysis. In this study, 4.5% was used as the baseline social discount rate that Korea Development Institute (KDI) has employed for evaluating public projects [15].

### 2.3. Cost Evaluation of Costs

In cost-benefit analysis, costs are divided into fixed and variable costs. The fixed costs for this study consisted of the price of the VCS2000 system and land price, and the variable costs consisted of operating expenses, including electrical costs, program operating costs, Internet charges, and facility maintenance costs (consumable item). Below Table 2 shows the total costs.

The VCS2000 system is used to estimate carcass yield after the slaughter process. To install the VCS2000 system, a space of 3 m × 5 m (15 m^2^) in a slaughterhouse is required [4]. The cost of the land for installing the system was determined by averaging the land price of 77 slaughterhouses accessed from publicly available information. The cost of the land was found to be 196,657 Korean won (KRW) per square meter (thus 2,949,855 KRW for 15 m^2^) [16].

The electricity cost was calculated using the formula in Equation (1). In the preliminary investigation, the electric consumption of the VCS2000 system was estimated to be about 200 W per hour, and the machine operating time was from 9 am to 12 pm and 1 to 6 pm, for a total of 8 h per day. The electricity cost was calculated based on the type of electricity used in slaughterhouses (“Industrial service (B); high-voltage A/option II”), under which more than 300 pigs per hour are slaughtered. The total annual power cost was found to be 3,514,490 KRW [17].

Annual electricity cost (KRW) = Demand charge [(8320 (KRW) × 8 h × 5 days × 52 weeks)] + Summer [{(6 h × 191.1 (KRW)) + (2 h × 109.0 (KRW))} × 5 days × 13 weeks] + Spring/Fall [{(2 h × 78.6 (KRW)) + (6 h × 109.0 (KRW))} × 5 days × 22 weeks] + Winter [{(5 h × 109.2 (KRW)) + (3 h × 166.7 (KRW))} × 5 days × 22 weeks](1)

Administrative costs for the VCS2000 program are incurred annually in the range of about 15% of total program costs (45,000,000 KRW). Therefore, the annual program operating cost for the VCS2000 program was estimated to be 6,500,000 KRW.

The VCS2000 system stores the image data obtained from slaughterhouses on its own server, which uses the Internet to transfer images. Prior to setting the Internet cost, the average Internet usage fee from three Korean mobile service companies (SKT, KT, and LGU+) was investigated. The average Internet rate for each mobile service company differed depending on the speed of the Internet, with an average monthly rate of 22,000 KRW for 100 Mbps, 33,000 KRW for 500 Mbps and 37,800 KRW for 1 Gbps. For this study, a 100 Mbps Internet connection was selected, and the annual cost was found to be 264,000 KRW [18,19,20].

Facility maintenance costs include RFID tag replacement costs and carcass number printer head replacement costs. It was determined that the cost of replacing RFID tags and printer heads would not occur during the three-year warranty period of the VCS2000 system, and that after three years, carcass number printer heads would be replaced once annually and RFID tags would be replaced 50 times annually. The VCS2000 distributors estimate that the carcass printer head replacement cost is 500,000 KRW each and RFID tags are 10,000 KRW each. For this study, the distribution price criteria of the company were applied, so the printer head cost for annual consumables was set at 500,000 KRW and the cost of RFID chips at 500,000 KRW.

The VCS2000 system cost 940 million KRW as of 2018. The fixed cost parameters in the VCS2000 project consist of the VCS2000 system price and land costs, which sum to 942,949,855 KRW. Operation cost consist of the electricity cost, consumable cost, program cost for operation, and the Internet cost. The electricity cost, cost for operation, and Internet cost are generated annually, however, consumable costs were considered to be generated after 3 years of operation because of the warranty period of the system. Therefore, these costs were included as consumable costs from the fourth year of the project onwards.

### 2.4. Depreciation Cost Calculation

Depreciation refers to the process of allocating an amount (the amount to be depreciated) less the residual value from the acquisition cost of an asset in a systematic and reasonable manner during each period in which the asset is in operation [21]. Depreciation costs were calculated using the declining balance depreciation method. The ‘declining balance depreciation’ is a method to account for a lot of depreciation costs at the beginning of a business. For calculating the depreciation are using investment, duration time, salvage value and the amortization factor (1-salvage value^1⁄(duration time)^). In this study, the duration of the depreciation cost calculation was set to 9 years, and the salvage value of the VCS2000 system after 9 years was set to 0.1%. The total investment was set as the facility investment cost (940 million KRW), excluding land cost, in the fixed cost category. The annual depreciation expense was calculated using the depreciation cost formula in Equation (2) below. In addition, the remaining value (940,000 KRW) was included in the 9th year’s cost.

(2) $$\mathrm{Depreciation}\;\mathrm{cost}\left(\mathrm{KRW}\right)=D_{n}=\left(\mathrm{Invest}-\overset{n-1}{\underset{i=1}{\sum }}D_{i}\right)\times {\left(1-\mathrm{salvage}\;\mathrm{value}\right)}^{\frac{1}{\mathrm{duration}\;\mathrm{time}}}$$

where ‘*D_n_*’ is the depreciation cost, ‘*n*’ is the total duration of the business, “Invest” is the VCS2000 investment price.

### 2.5. Evaluation of Benefits

The benefits were separated into direct and indirect benefits. Direct benefits include those which reduce labor costs while indirect benefits are those from meat yield improvement and the effect of optimizing feed costs and management. Further definitions of the benefits defined for this study are shown in Table 3.

#### 2.5.1. Direct Benefit

The benefits of reducing labor costs that correspond to direct benefits were set based on the Korea minimum hourly wage in 2021. In conventional slaughterhouses, workers must classify carcasses in chilling rooms directly at the time of shipment of pig carcasses. However, with the introduction of the VCS2000 system, it is possible for slaughterhouse chilling room personnel to be employed in other tasks, resulting in an alternative benefit of one person. In addition, the introduction of the system can replace carcass numbering (which was manually carried out by workers) with the use of a carcass number printer in the VCS2000 system. Currently, the introduction of mechanical automatic judging machines by the Livestock Products Quality Evaluation Institute does not mean the complete replacement of grading personnel, and only has the function of enhancing the work efficiency of the quality appraiser. Therefore, in this study, the replacement of the staff of the Livestock Products Quality Assessment Service was considered to be zero. At this point, the introduction of the VCS2000 system will allow for the replacement of one chilling room manager and one staff member employed to record carcass numbers, thus reducing labor costs for a total of two workers. In calculating benefits for reducing labor costs, 8720 KRW was applied to each worker monthly salary (i.e., the minimum hourly wage in 2021) and 209 working hours were applied per month. Accordingly, the minimum monthly salary was calculated as 1,822,480 KRW. Thus, it can be expected that the replacement of one worker will result in an annual economic benefit of 21,869,760 KRW. As noted previously, the introduction of the VCS2000 system will affect two workers, with a total benefit calculated to be 43,739,520 KRW [22].

#### 2.5.2. Indirect Benefit

Indirect benefits from the VCS2000 system result from improving the quantity of LMP and the consequent effect of reducing feed costs related to feeding and management. Because the benefits of improving the quantity of LMP, and reducing feed costs related to feeding and management, are benefits from the target slaughterhouses using the VCS2000, a prediction of the number of slaughters per year is required. The slaughter number used for the economic analysis was 469,000 carcasses per annum [23]. This figure was derived based on the average annual slaughter volumes from 7 slaughterhouses (i.e., Minsok LPC, Honju meat, Mokwoochon Gimje meat factory, Pukyong livestock products factory and Jeju LPC, Hyupsin meat LPC, NH Daejeon-Chungnam Porkvill), which were using the VCS2000 system as of 2020 (see Table 4 for more details). Before calculating the benefits due to increasing the quantity of meat, the speed of improvement of the breeding stock to increase the quantity of meat per year must be defined; however, no improvement in LMP (%) is currently being made in Korea. Therefore, the speed of genetic improvement in this study was based on data from DanBred International, Herlev, Denmark [24]; thus, the annual rate of genetic improvement of finishing pig LMP was 0.10% per year. In the second quarter of 2019, the Hongju Meat Co. Ltd. graded a total 262,482 carcasses, for which their LMP values averaged about 55.9%, and their average weight was about 88 kg. If the LMP of a pig with a carcass weight of about 88 kg increases by 0.10% per year, the increase in meat can be calculated to be about 88 g; this value was included in the formula, that is, the improvement in meat quantity achieved using the data from the VCS2000 system to improve breeding practices was set to 88 g of lean meat per annum per carcass. Besides, even if farms ship livestock to slaughterhouses where VCS2000 is installed, it is judged that not all farms use VCS2000 data for breeding or feed optimization. Therefore, in this analysis, the number of farms shipped to the slaughterhouse with VCS2000 installed was estimated, and the farms using meat quantity data for breeding among the farms were defined as the leading farmers, and the benefits generated for the leading farms were defined. First, to calculate this, it is necessary to calculate the number of farmers applying for slaughter at one slaughterhouse. According to 2020 statistics, the total number of pig breeding farms nationwide was 6078 farms, and the total pig slaughter volume in 2020 was 18,329,952 pigs [23,25]. By dividing the number of pigs slaughtered per year by the number of farms, the average number of pigs shipped per farm can be derived. Although it is judged that there is a difference in the number of annual shipments depending on the size of the farm, it is calculated that about 3016 pigs are shipped annually on average per one farm. The average slaughter volume of the VCS2000 installed slaughterhouse is calculated to be about 469,000 heads (Table 4), and when it is judged that 3016 pigs are shipped per farm. It can be calculated that approximately 156 farms are shipped to the slaughterhouse where the VCS2000 system was installed. It was determined that 10% of the leading farmers used meat yield data for farm management. The number of leading farms was set at 16 (first decimal place rounding applied) and 3016 units per farm were determined to be annually shipped, which resulted in benefits for 48,256 pigs per year (Equation (3)), which were used in benefit calculations (4) and (5). The formula for the benefit of the rate of increase in the LMP of meat is shown in Equation (3). The benefits predicted by this formula were calculated to be 32,951,565 KRW per annum. Furthermore, due to the increase in the LMP index of 0.1% per annum, an additional increase of 32,951,565 KRW per year was calculated.

Leading farms slaughtering amounts per year (48,256 carcasses) = Leading farms number (16 farms) × pigs shipped amount per one farm during one year (3016 animals)(3)

The benefits of increasing the LMP (KRW) = 48,256 carcasses (leading farms slaughtering amounts per year) × 798.4 KRW (pork meat price per 100 g (on 2020)) × 0.88 (increasing meat yields due to 0.1% annual increasing in LMP (88 g))(4)

The benefit associated with reducing production costs by customized feeding relates to the effect of reducing feed cost through feeding and management. Feed prices are highly affected by the price of grain, which is a raw material. Feed companies are unable to quickly change grain selling prices because cheap grain has not been proven to be effective for livestock in terms of its nutritional aspect. However, producers can quickly respond to changes in global grain prices as they can easily check the effects on the amount of meat produced due to changes in feed stock when VCS2000 data is used for feeding and management. As a result, production costs are expected to be reduced due to customized feed benefits by region, farm, and feeding and management stages. In particular, benefits are expected to be derived from the appropriate feed used during the growing-fattening period; the formula used to calculate this benefit is defined in Equation (5).

The benefits of reducing pig feed consumption through optimization (KRW) = 110 day (Total feeding periods) × 3 kg/day (pig feeding consumption per day) × 48,256 carcasses (predicted carcass amount per year in leading farms) × 10 KRW (price differences of grain feed)(5)

The growing-fattening period is 110 days and the feed consumption per pig was set at 3 kg per day. Finally, it was calculated that the benefits of reducing production costs of feed under this formula will result in an annual cost reduction of 154,770,000 KRW.

### 2.6. Benefit/Cost Ratio (BCR) Analysis

BCR refers to the ratio of the present value of the benefit flows arising from the investment divided by the present value of the cost flows. If this ratio is greater than 1 (BCR ≥ 1), the project is evaluated to be economically feasible for investment [26]. The calculation of BCR is shown Equation (6).

(6) $$\mathrm{BCR}=\overset{N}{\underset{t=0}{\sum }}\frac{NPB_{t}}{{\left(1+r\right)}^{t}}÷\overset{N}{\underset{t=0}{\sum }}\frac{NPC_{t}}{{\left(1+r\right)}^{t}}\;$$

where *t* is the cash flow period (years), *N* is the total duration of the business, *r* is the discount rate, *NPB_t_* is the net present benefit at time *t*, and *NPC_t_* is the net present cost at time *t*.

### 2.7. Net Present Value (NPV) Analysis

Net present value (NPV) analysis is commonly used in evaluating the feasibility of a project. Because the costs and benefits arising from a project continue beyond the present and into the future, the future benefits and costs should be considered by converting them to the present values. In other words, NPV refers to the present value of the expected future values from the investment [27]. The formula for obtaining NPV is as shown in Equation (7).

(7) $$\mathrm{NPV}=\overset{N}{\underset{t=0}{\sum }}\frac{C_{t}}{{\left(1+r\right)}^{t}}$$

where *t* is the duration of the cash flow (in years), *N* is the duration of the business/project, *r* means the discount rate, and *C_t_* means the net present cash flow (benefit-cost) at the time *t*. According to NPV calculations, if NPV is greater than zero (NPV > 0), it is determined that there is an investment value, and if it is less than zero, it is assessed that there is no investment value.

### 2.8. Internal Rate of Return (IRR) Analysis

Internal rate of return is defined as the discount rate (r) that makes the net present value (NPV) zero. IRR refers to the average annual return on an investment over the duration of the project. If the IRR is greater than the discount rate (r) (IRR > r), the investment is considered to be economically valuable. The calculation of IRR is shown in Equation (8). where *t*, *N*, *r*, and *C_t_* are defined as in Equation (6).

(8) $$\overset{N}{\underset{t=0}{\sum }}\frac{C_{t}}{{\left(1+r\right)}^{t}}=0$$

### 2.9. Sensitivity Analysis

The calculated benefits and costs in the cost-benefit analysis include many assumptions and uncertainties. In cost-benefit analysis, sensitivity analysis is performed to address these uncertainties. Sensitivity analysis identifies changes in NPV, IRR, and B/C ratio by changing the critical variables that can affect economical value within a certain range. For this study, the social discount rate, total cost, and total benefit were set as the main variables that were likely to affect the results of the cost-benefit analysis; thus, each of the three parameters was set as an individual variable and used for analysis. Each variable was evaluated for changes in NPV, IRR, and B/C ratio based on a change of ±10%. Furthermore, sensitivity analysis was also conducted on the assumption of changes in the annual number of slaughtered pigs, changes of the genetic improvement speed of annual LMP, changes in the price of feed and changes of farm numbers using meat yield data.

## 3. Results and Discussion

### 3.1. Economic Analysis Results

The annual cash flow and economic analysis results from the cost-benefit analysis shown in Table 5. This cost-benefit analysis is represented by the NPV. NPV, B/C ratio, and IRR by applying the machine’s persistent period of 9 years and social discount rate of 4.5%. In the starting year of the VCS2000 project, the highest cost was incurred by the purchase of the machine, and it can be seen that benefits did not occur immediately. The operating cost remained constant from the first year, before increasing in the fourth year of the business as the VCS2000 quality assurance period (three years) came to an end. The expense increase starting from the fourth year was incurred due to the additional costs in repair and maintenance for the system. In the first year, the benefits of reducing the number of workers (by two people) due to the introduction of the VCS2000 system were realized, and from the third year of the project, the benefits of reducing feed costs and increasing the amount of meat due to the improvement of pigs’ LMP (%) increased. The total present value of benefits arising from the VCS2000 is calculated to be approximately 1.55 billion KRW, the total present value of expenses is 0.94 billion KRW, and the NPV is calculated to be approximately 615 million KRW. In addition, since the B/C ratio, which is the net present benefit divided by the net present cost, has a value greater than 1, the benefits resulting from installing VCS2000 are greater than the costs required to introduce and operate the system. The IRR value was 13.52%, which is greater than the discount rate (4.5%), indicating that the economic value of the device to determine pork carcass meat yields is sufficient.

### 3.2. Sensitivity Analysis

**Figure 2:**
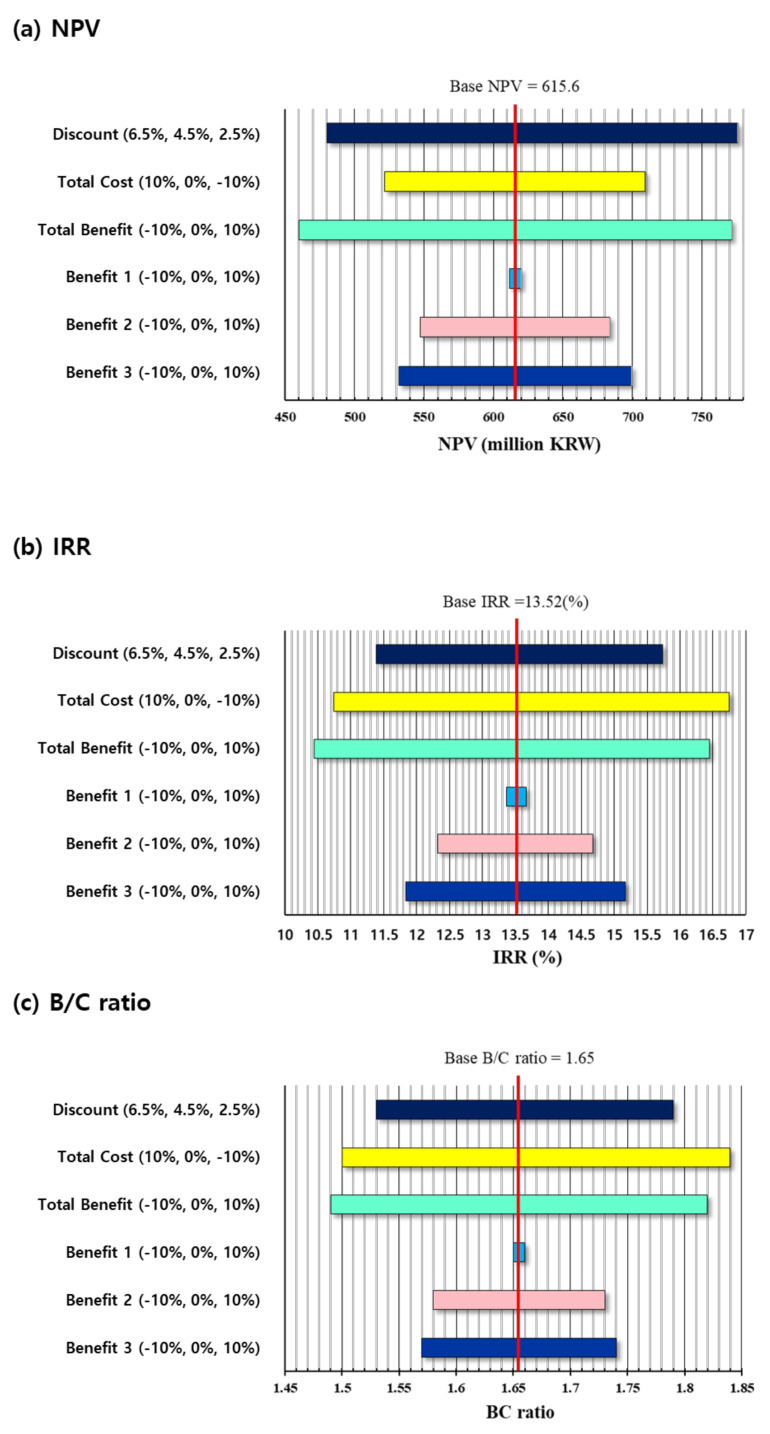
Sensitivity analysis result of each parameter. (**a**) net present value (NPV) results; (**b**) internal rate of return (IRR) results; (**c**) benefit/cost (B/C) ratio results.

Figure 2 shows the results of the sensitivity analysis of the VCS2000 system. In the sensitivity analysis, the social discount rate, total benefit, total cost, and each benefit were adjusted within a certain range for analysis. When analyzing the sensitivity, the social discount rate (4.5%) was altered within a range of ±2% to examine the effect on the B/C ratio, NPV, and IRR. As the social discount decreases, the B/C ratio, NPV, and IRR increase due to an increase in the present value of future benefits. Conversely, as the discount rate increases, it can be seen that the present value of future benefits decreases and the B/C ratio, NPV, and IRR decrease. To determine the effect on the analysis of a change in the cost of the VCS2000 system, the effects of a ±10% change in cost on the B/C ratio, NPV, and IRR were examined. As the cost decreased by 10%, the net present value (NPV) increased from the existing 615.6 million KRW to 709.5 million KRW, and when the cost increased by 10%, the net present value decreased to 521.8 million KRW. It can be seen that there is a difference of about 187.7 million KRW when the total cost increased by 10% compared to when it decreased by 10%.

To investigate the effect of the total benefit of the VCS2000 and the result of the cost-benefit analysis when each benefit fluctuates, the total benefit was increased or decreased by 5% within a range of ±10% based on the total benefit and each benefit. As the total benefit increased by 10%, the NPV increased to 771.3 million KRW, and when the total benefit decreased by 10%, it fell to 459.9 million KRW.

VCS2000’s cost-benefit analysis was conducted to check how each benefit affects the overall analysis when each benefit item was adjusted by 5% in the range of ±10%. Benefit item 1 shows the case when two workers are removed while the system is in operation. When benefit item 1 increased or decreased by 10%, the NPV increase rate was about 8.3 million KRW. Benefit item 2 refers to the benefit obtained when the amount of meat raised increases by 88 g each year when carcass grading data from VCS2000 is used for breeding. Benefit item 2 changed the NPV by about 136 million KRW when the benefit increased or decreased by ±10%. Benefit item 3 refers to the impact of reducing the cost of feed to pigs in terms of feeding and management costs. When benefit item 3 was increased by 10%, the NPV was about 699.2 million KRW, and when it was decreased by 10%, the NPV was 532.1 million KRW. Benefit item 3 showed an NPV change of approximately 167.1 million KRW as the benefit increased or decreased by 10%.

As a result of the sensitivity analysis, it was confirmed that changes in the ‘total benefit variance’ had more influence on the NPV than other variables. In the case of discount rate change, the variation of the NPV was not large, and the maximization of the benefits was judged to have a significant effect on the NPV. When analyzing the sensitivity of each benefit, the benefits impacts were ranked in the order of “reduced feed cost benefit (benefit 3)”, “benefit of increasing meat amount (benefit 2)”, and “benefit of reduction in manpower (benefit 1)”. The benefits of manpower reduction did not significantly affect the sensitivity analysis because the portion of manpower is relatively low in comparison with the other factors such as breeding/feeding practices and meat yield increment as a result of using the VCS2000 system.

#### 3.2.1. Sensitivity Analysis According to Slaughter Volume Change and Meat Content Enhancement

Calculating the annual number of slaughters is an important indicator for benefit calculation. For calculating “Benefit of improving the lean meat percent (benefit item 2)” and “Benefit of reducing feed costs based on breeding management (benefit item 3)”, this study used the average slaughtering amount (469,000 carcasses) in 2020 at seven slaughterhouses. The average slaughter numbers of the seven slaughterhouses were used for analysis, but the total slaughter count at Jeju LPC in 2020 was 493,227 head, and at the Moguchone Gimje meat processing plant 743,201 head, both of which were higher than average (Table 4). This was therefore reflected in the sensitivity analysis as the change in the NPV, IRR, and B/C ratio as the annual slaughter volume changed to 240,000 heads (1543 heads pre one farm), 400,000 heads (2572 heads pre one farm), 560,000 heads (3601 heads pre one farm), and 740,000 heads (5273 heads pre one farm) (see Equation (3)). In addition, no guideline exists for quantifying the increase in the amount of meat in the current domestic standards for pig breeding improvement. Thus, it is difficult to accurately predict the annual increase in meat amounts due to the improvement of the breeding stock. In this study, sensitivity analysis was undertaken by setting the annual increase in the weight of the LMP at 0 g (0.0%), 44 g (0.05%), 88 g (0.10%), 132 g (0.15%), and 176 g (0.20%). The results of sensitivity analysis according to changes in annual slaughter volume and enhancement of annual LMP (%) content are shown in Table 6. It can be seen that the NPV is −124.3 million KRW when the annual LMP growth rate is 88 g and the number of slaughtered pigs is 240,000, while the NPV increases to 909.7 million KRW at 560,000 head and 1.49 billion KRW at 740,000 head. This can be attributed to the fact that the amount of data acquired and accumulated increases as the number of slaughters increases when using VCS2000, and thus the number of farms receiving feedback increases. When analyzing the sensitivity to the increase in the annual LMP, the annual slaughter volume is 390,000 head and the NPV is 6.98 billion KRW.

#### 3.2.2. Sensitivity Analysis by Stepwise Feed Price Change and Annual Slaughter Volume Change

When VCS2000’s meat yield measurement data are used in the precision feeding and management of pigs, it is possible to obtain a feed cost reduction effect by using appropriate feed amounts for each breeding stage. When analyzing economic feasibility in this study, the effect of reducing the feed cost was considered to be 10 KRW per pig; however, this value may change due to the volatility of the global grain market. Therefore, in this study, the sensitivity was analyzed by changing the feed price by ±10 KRW. In addition, the difference in the number of slaughtered pigs was also adjusted to 240,000 heads (1543 heads pre one farm), 400,000 heads (2572 heads pre one farm), 560,000 heads (3601 heads pre one farm), and 740,000 heads (5273 heads pre one farm) to investigate changes in the B/C ratio, NPV, and IRR. Table 6 shows the results of the sensitivity analysis in which feed price for each stage and the annual slaughter volume were varied.

The NPV was expected to be −319.6 million KRW for a slaughterhouse of the annual slaughter volume of 400,000 pork carcasses with the condition of zero feed savings. When feed savings increased to 20 KRW, NPV increased by about 2.5 times compared to the existing NPV of 392.7 million won at 10 KRW. In addition to these effects, the savings of feed costs were also found to affect the NPV. Therefore, when applying VCS2000 data to feed formulations, it is expected that feed companies will be able to help in finding feed ingredients that have excellent effects at an appropriate price.

## 4. Future Directions

A system for judging pig carcass meat yields is not easy to apply due to the high price of the machinery. However, it is judged that the utility value of this automatic judgment machine is high because of the utilization value of the data it generates. To use the machine effectively, research on the selection of breeding pigs, and optimization of feeding and management using VCS2000 meat yield data is needed. Customized breeding pig research and development using the amount of meat calculated by the carcass meat yield judgement machine is a form of smart farm technology in which accumulated butchering data by cut is used during the production stage.

To effectively connect a smart farm to the VCS2000 system, individual pig information should be automatically gathered using object recognition technology on farms. Thus, it is necessary to develop technology that can automatically recognize individual pigs through artificial intelligence-based imaging or RFID technology, and periodically assess the type and amount of feed consumed. This data, from the breeding stage until slaughter, can be integrated and managed in the cloud server.

To fully automate grading, automatic judging technology for carcass meat quality and defects other than meat yield is needed. Europe, which is the home of the world’s leading companies that produce carcass meat yield judging machines, focuses on meat yields rather than machines that judge meat quality or identify carcass defects. Therefore, it is desirable that a device to judge meat quality and defects based on domestic grading criteria is developed with domestic technology.

An automatic carcass judging machine, operated using digital image analysis, can be used to analyze the position of muscles and bones, in addition to determining grades. Moreover, it can provide sophisticated anatomical information about the carcass to enable deboning by robots in the future. Thus, when unmanned automation technology is expanded to include the livestock industry, automatic deboning robots will help solve the problem of the shortage of manpower for slaughter work, and dramatically improve butchering productivity and economic feasibility.

## 5. Conclusions

This study was conducted to identify means of increasing the economic value and utility of the VCS2000, an automatic grading machine, through cost-benefit analysis. In the analysis, based on internal data from the Korea Institute for Animal Products Quality Evaluation, the cost and benefit factors involved in the operation of the VCS2000 were set and each figure was derived. The detailed items of the costs and benefits were quantified for economic analysis and the net present value (NPV), the benefit–cost ratio (B/C ratio), and the internal rate of return (IRR) were calculated.

During the business period of the VCS2000, the net present value was found to be approximately 615 million KRW, the B/C ratio was 1.65, and the IRR was 13.52%. In each of the economic assessments undertaken, the analysis showed that the benefits outweigh the costs in the long term. In particular, the investment will pass the break-even point within seven years of the project due to the benefits of increasing LMP (%) and reducing feed costs. To maximize the economic effects of the VCS2000, the efficiency of the machine should be enhanced by increasing the number of pigs that are slaughtered using the automatic grading machines of slaughterhouses, and judging data of meat yields for each area should be provided to breeding farms and used in breeding.

Due to the increase in the automation of modern-day slaughter-houses around the world, systems such as the VCS2000, which have a high initial cost, can be seen as a big hurdle for slaughter companies. However, since the reduction in labor cost alone cannot justify the cost of the system if benefits 2 and 3 of our economic analysis are not realized, maximizing the use of the system, especially in the optimization of breeding practices and feeds, can help to alleviate the burden of the initial cost.

This study proves that the automation of the slaughter line (or part of the slaughter line) is economically viable and thus can be employed for modernizing meat production in Korea. However, due to the fact that this technology (specifically the VCS2000 system) is developed by companies outside of Korea, the initial cost of the system and the repair and maintenance costs are relatively high. If these technologies are developed domestically, the initial cost, and repair and maintenance costs, can be reduced, thus rendering the deployment of such technologies even more economically beneficial.

## Figures and Tables

**Table 1:** VCS2000 resources and scopes of cost-benefit analysis.

Classification	Contents	
Analysis Target	VCS2000	
Analysis of documents and frame of analysis	Cost	VCS2000 system cost: 940,000,000 KRW
		Land cost: 2,949,855 KRW
		Management periods 1–3 years: 9,188,636 KRW/year Management periods 4–9 years: 10,688,636 KRW/year
	Benefit 1	Benefit of reduction for labor cost: 43,739,520 KRW/year
	Benefit 2	Benefit of increase meat yields (year per 88 g): 32,951,565 KRW/year
	Benefit 3	Benefit of feeding cost reduction: 154,770,000 KRW/year
Analysis methods	Net present value (NPV), Internal return of rate (IRR), benefit-cost ratio (B/C ratio)	
Period of analysis	Cost input period: 9 years Benefit 1 generation period: Occurs on the 1st year of business Benefit 2 generation period: Occurs from the 3rd year of business Benefit 3 generation period: Occurs from the 3rd year of business (Benefit 2 continued increase in annual lean meat amount by 88 g since the 3rd year)	
Discount ratio	4.5% (Republic of Korea Ministry of economy and finance, 2019)	
Sensitive analysis	Sensitive analysis 1: sensitive analysis changes about discount ratio changing discount ratio: 2.5~6.5% ranges; 0.5%	
	Sensitive analysis 2: sensitive analysis changes about total cost Changing cost ratio: ±10% ranges; 5% variation	
	Sensitive analysis 3: sensitive analysis changes about total benefit (±10% range; 5% variation)	
	Sensitive analysis 4: Sensitive analysis of individual benefit variation (±10% range; 5% variation)	
	Sensitive analysis 5: Sensitivity Analysis according to changes of the annual slaughtering volume and the Improvement of lean meat percent. Annual slaughtering amount: 240,000, 400,000, 560,000, 740,000 Annual lean meat content increasing amount range: 0–176 g (±44 g)	
	Sensitive analysis 6: Sensitivity analysis according to changes in feed price and annual slaughtering volume. Feed price difference by growth stages: 0, 5, 10, 15, 20 (±5 KRW) Annual slaughtering amount: 240,000, 400,000, 560,000, 740,000	

**Table 2:** Direct and indirect benefits and calculation formula.

Classification	Category		Sub-Category and Calculation Formula	Expense (KRW)
Direct benefit	Benefit 1	Labor cost reduction	Minimum wage on 2021(1,822,480 KRW) × 12 month × number of personnel reductions (2 persons) = 21,869,760	43,739,520
Indirect benefit	Benefit 2	Benefit of improving the lean meat percent	798.4 KRW (price per 100 g on 2020) × 48,256 carcasses (expected slaughtering amounts of leading farm) × 0.88 g (Increasing of lean meat amounts according to an annual increase of 0.10% LMP)	32,951,565
	Benefit 3	Benefit of reducing feed costs based on breeding management	110 day (growing fattening period days) × 3 kg/day (amount of intake) × 10 KRW/kg (difference of feeding price by different breeding step) × 48,256 carcasses (expected slaughtering amounts of leading farm)	154,770,000
Total direct benefit amounts				43,739,520
Total indirect benefit amounts				187,721,565
Total benefit amounts				231,461,085

KRW: Korean won.

**Table 3:** List of slaughterhouses installed ‘VCS2000’ and their slaughtering amount in 2020.

Company	Year of Installation	Slaughtering Amount in 2020
Minsok LPC Co, Ltd.	2016	254,028
Hongju Meat Co, Ltd.	2018	478,498
NH Moguchon Gimje Meat Processing Factory	2018	743,201
Pukyong Livestock Products Co., Ltd.	2019	380,344
Jeju LPC	2019	493,227
Hyupsin Meat LPC	2020	174,625
NH Daejeon-Chungnam Porkvill	2020	384,869
Total number		2,908,792
Average		415,541

**Table 4:** Results of cash flow and cost-benefits analysis.

Year	Benefit (B)	Cost (C)	Depreciation Cost	Net Present Benefit (NPB)	Net Present Cost (NPC)
Operating day	0	0	0	0	0
1 year	43,739,520	12,138,491	503,690,650	41,856,000	515,829,141
2 year	0	9,188,636	233,792,489	0	242,981,125
3 year	187,721,565	9,188,636	108,516,861	164,499,770	117,705,497
4 year	220,673,130	10,188,636	60,369,065	185,047,956	50,781,347
5 year	253,624,694	10,188,636	23,379,249	203,521,401	26,936,584
6 year	286,576,259	10,188,636	10,851,686	220,060,688	16,156,774
7 year	319,527,824	10,188,636	5,036,906	234,798,138	11,188,162
8 year	352,479,389	10,188,636	2,337,925	247,858,264	8,808,491
9 year	385,430,954	11,128,636	1,085,169	259,358,195	8,218,723
Total	2,006,033,814	92,587,579	939,060,000	1,557,000,412	941,356,505
Results	NPV = 616 million KRW, B/C ratio = 1.65, IRR = 13.52%				

**Table 5:** Sensitivity analysis according to annual slaughter volume difference and annual lean meat percentage change.

Annual Lean Meat Increasing Amounts (LMP%)	Annual Slaughtering Numbers											
	240,000 (YP: 1543 Pigs)			400,000 (YP: 2572 Pigs)			560,000 (YP: 3601 Pigs)			740,000 (YP: 4758 Pigs)		
	B/C Ratio	NPV (mili)	IRR (%)	B/C Ratio	NPV (mili)	IRR (%)	B/C Ratio	NPV (mili)	IRR (%)	B/C Ratio	NPV (mili)	IRR (%)
0 g (0%)	0.50	−472.2	−17.65	0.80	−187.2	−5.97	1.10	97.7	2.80	1.44	418.1	10.98
44 g (0.05%)	0.68	−298.2	−9.34	1.11	102.7	2.72	1.54	503.7	11.90	2.01	954.5	20.50
88 g (0.10%)	0.87	−124.3	−3.45	1.42	392.7	9.12	1.97	909.7	18.76	2.58	1490.9	27.81
132 g (0.15%)	1.05	49.6	1.25	1.73	682.6	14.34	2.40	1315.6	24.42	3.15	2027.3	33.92
176 g (0.20%)	1.24	223.6	5.22	2.03	972.6	18.81	2.83	1721.6	29.32	3.72	2563.7	39.24

mili: Million Korean won; YP: Yearly pig shipments per one farm.

**Table 6:** Sensitivity analysis according to adjusting feed price difference and slaughter volume change by growth stage.

Feed Price Difference	Annual Slaughtering, Number of Pig Carcasses											
	240,000 (YP: 1543 Pigs)			400,000 (YP: 2572 Pigs)			560,000 (YP: 3601 Pigs)			740,000 (YP: 4758 Pigs)		
	B/C Ratio	NPV (mili)	IRR (%)	B/C Ratio	NPV (mili)	IRR (%)	B/C Ratio	NPV (mili)	IRR (%)	B/C Ratio	NPV (mili)	IRR (%)
0	0.41	−551.6	−18.06	0.66	−319.6	−8.73	0.91	−87.6	−2.11	1.18	173.3	3.76
5	0.64	−337.9	−10.01	1.04	36.6	0.91	1.44	411.0	9.07	1.88	832.1	16.57
10	0.87	−124.3	−3.45	1.42	392.7	9.12	1.97	909.7	18.76	2.58	1490.9	27.81
15	1.09	89.4	2.34	1.80	748.8	16.43	2.50	1408.3	27.47	3.28	2149.8	37.99
20	1.32	303.0	7.54	2.17	1104.9	23.10	3.03	1906.9	35.47	3.98	2808.6	47.39

mili: Million Korean won; YP: Yearly pig shipments per one farm.

## Data Availability

The data are available on request from the corresponding author.
